# Enhancing geriatric trauma mortality prediction: Modifying and assessing the Geriatric Trauma Outcome Score with net benefit and decision curve analysis

**DOI:** 10.1111/acem.15103

**Published:** 2025-02-06

**Authors:** Pawan Acharya, Tabitha Garwe, Sara K. Vesely, Amanda Janitz, Jennifer D. Peck, Alisa M. Cross

**Affiliations:** ^1^ Department of Biostatistics and Epidemiology University of Oklahoma Health Sciences Center Oklahoma City Oklahoma USA; ^2^ Division of Trauma and Acute Care Surgery, Department of Surgery University of Alabama at Birmingham Birmingham Alabama USA; ^3^ Department of Surgery University of Oklahoma Health Sciences Center Oklahoma City Oklahoma USA

**Keywords:** decision curve, geriatric trauma, net benefit, prediction

## Abstract

**Objective:**

Calibration and discrimination indicators alone are insufficient for evaluating the clinical usefulness of prediction models, as they do not account for the cost of misclassification errors. This study aimed to modify the Geriatric Trauma Outcome Score (GTOS) and assess the clinical utility of the modified model using net benefit (NB) and decision curve analysis (DCA) for predicting in‐hospital mortality.

**Methods:**

The Trauma Quality Improvement Program (TQIP) 2017 was used to identify geriatric trauma patients (≥ 65 years) treated at Level I trauma centers. The outcome of interest was in‐hospital mortality. The GTOS was modified to include additional patient, injury, and treatment characteristics identified through machine learning methods, focusing on early risk stratification. Calibration and discrimination indicators, along with NB and DCA, were utilized for evaluation.

**Results:**

Of the 67,222 admitted geriatric trauma patients, 5.6% died in the hospital. The modified GTOS score included the following variables with associated weights: initial airway intervention (5), Glasgow Coma Scale ≤13 (5), packed red blood cell transfusion within 24 h (3), penetrating injury (2), age ≥ 75 years (2), preexisting comorbidity (1), and torso injury (1), with a total range from 0 to 19. The modified GTOS demonstrated a significantly higher area under the curve (0.92 vs. 0.84, *p* < 0.0001), lower misclassification error (4.9% vs. 5.2%), and lower Brier score (0.036 vs. 0.042) compared to the original GTOS. DCA showed that using the modified GTOS for predicting in‐hospital mortality resulted in higher NB than treating all, treating none, and treating based on the original GTOS across a wide range of clinician preferences.

**Conclusions:**

The modified GTOS model exhibited superior predictive ability and clinical utility compared to the original GTOS. NB and DCA offer valuable complementary methods to calibration and discrimination indicators, comprehensively evaluating the clinical usefulness of prediction models and decision strategies.

## INTRODUCTION

Geriatric (age ≥65 years) trauma constitutes 8.5% of all geriatric patient care.^1^ According to an analysis of the National Trauma Database in the United States, the proportion of geriatric trauma cases, relative to total trauma cases, increased from 18% to 30% between 2005 and 2015.^2^ Aging not only heightens the risk of traumatic injury but also exacerbates the severity and mortality risk associated with trauma due to prevalent comorbidities, polypharmacy, and diminished physiological reserves.^3^, ^4^ Despite the disproportionate burden of morbidity and mortality, geriatric trauma patients are often undertriaged to higher level trauma centers, resulting in delayed care and poorer prognosis.^5^, ^6^

The increasing proportion of geriatric trauma also intensifies the complexity of trauma care.^7^, ^8^, ^9^ With a higher volume of patients presenting with complex cases, it is essential to identify those at higher risk of death to promptly decide on a tailored course of action. One such model developed for risk stratification is the Geriatric Trauma Outcome Score (GTOS). This model incorporates age, Injury Severity Score (ISS), and blood transfusion within 24 h of admission to predict in‐hospital mortality.^10^ In a multicenter external validation study, Cook et al.^10^ found that the GTOS model had a misclassification error of 9.97% and 9.79%, an area under the receiver operating characteristics curve (AUC) of 0.86 (95% confidence interval [CI] 0.86–0.87) and 0.82 (95% CI 0.81–0.83) in the validation and derivation samples, respectively. They concluded that the GTOS model accurately predicts in‐hospital mortality in injured elderly patients.

The GTOS model, however, does not incorporate several key clinical variables that are critical for accurately predicting outcomes in geriatric trauma patients. Physiologic indicators, such as the Glasgow Coma Scale (GCS) score and the need for airway intervention, provide vital insights into a patient's initial neurologic and respiratory stability, essential for early risk assessment. Injury characteristics, including penetrating trauma and torso injuries, offer additional context by capturing mechanisms and anatomical locations strongly associated with adverse outcomes. Furthermore, preexisting comorbidities and advanced age, particularly age ≥ 75 years, highlight patient‐specific vulnerabilities such as frailty and diminished physiological reserves, which can significantly complicate trauma care. Excluding well‐established predictors like injury type, initial vital signs in the emergency department (ED), initial interventions (such as airway support), and preexisting comorbidities could limit the GTOS model's prediction accuracy for geriatric trauma outcomes.^6^, ^11^, ^12^ Additionally, assigning an ISS to a patient often requires advanced imaging or time‐consuming procedures, which may limit its usefulness for early prediction of outcomes.^13^ Integrating these factors into the framework for evaluating geriatric trauma outcomes addresses critical limitations of the GTOS model by accounting for both acute and chronic dimensions of risk.

While prediction models are generally assessed using calibration and discrimination measures, such as the AUC,^14^ the AUC has limitations because it does not account for the clinical consequences of misclassification.^15^ In trauma risk assessment, missing a major trauma patient at high risk of death (false negative) has far greater consequences than incorrectly labeling a low‐risk patient as high risk (false positive). This is reflected in accepted thresholds for undertriage (<5%) and overtriage (<35%) rates.^16^ Incorporating the differential costs of these misclassifications into prediction model enhances their clinical utility. Decision curve analysis (DCA)^17^, ^18^, ^19^, ^20^ provides an effective method for evaluating a model's net clinical benefit (NB), offering insights into its practical value beyond AUC metrics, and aiding in the identification of optimal strategies for geriatric trauma care.

The primary objective of this study was to modify the existing GTOS model and, secondarily, to evaluate and compare the clinical utility of the modified model to the original GTOS using DCA. We hypothesize that in‐hospital mortality among injured older adults can be better predicted by identifying preexisting comorbidities, specific injuries, vital signs on arrival, and early interventions that may increase the risk of short‐term mortality.

## PATIENTS AND METHODS

### Study design and population

**FIGURE 1:**
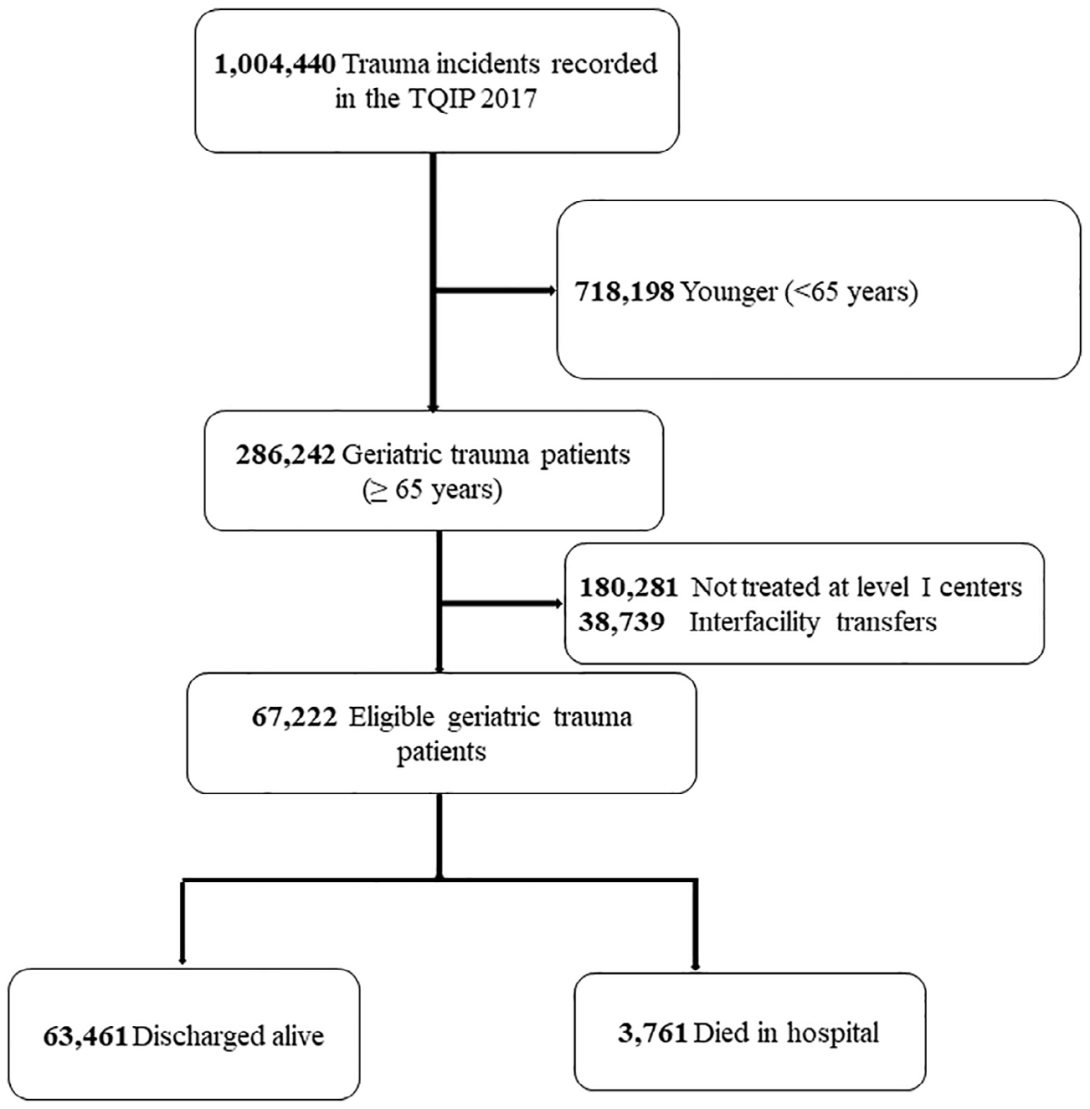
Exclusion and inclusion criteria. TQIP, Trauma Quality Improvement Program.

This retrospective prognostic study utilized the 2017 Trauma Quality Improvement Program (Trauma Quality Improvement Program [TQIP] 2017) dataset.^21^ Included were 67,222 geriatric patients (≥ 65 years) directly admitted and treated at Level I trauma centers. Figure 1 shows the flow of patient selection.

### Study outcome

The outcome of interest was in‐hospital mortality, defined as death occurring before discharge from the hospital.

### Predictor variables

Since this study aimed to modify and assess the utility of the GTOS model, age and transfusion of packed red blood cells (pRBC) within 24 h of admission variables were selected a priori for the modified model. The ISS variable was only used to replicate the GTOS model as per the original study: GTOS = Age (years) + (2.5 × ISS) + 22 (if given any pRBC in the first 24 h after injury).

To develop a predictive model and subsequently create a modified GTOS risk score, patient demographic characteristics such as sex, body mass index (BMI), race, and ethnicity were included as candidate predictor variables. The injury characteristics considered were injury type, injury mechanism, and specific injuries based on the International Classification of Diseases, Tenth Revision (ICD‐10), codes or the Abbreviated Injury Scale body region. Incorporating both injury type and injury mechanism variables is crucial, as each provides unique and independent insights into trauma outcomes. Injury type, which categorizes the nature of the trauma (such as blunt or penetrating), and injury mechanism, which identifies the specific cause (e.g., falls, motor vehicle collisions), both influence the severity, treatment approach, and prognosis of the patient. Prehospital EMS transportation was also analyzed. Initial ED vital signs including systolic blood pressure (SBP), heart rate (HR), Shock Index (SI), respiratory rate (RR), and total GCS were considered in the analysis both as continuous and binary variables (defined based on clinically acceptable thresholds, such as SBP ≤110 or ≥200, RR <10 or RR ≥24, HR ≤60 or ≥110, GCS ≤13).^22^ The SI was calculated by dividing HR by SBP, and a threshold of 0.9 was used to define a binary SI variable. Other clinical factors considered included hospital interventions, including initial ED oxygen supplementation, airway support (airway insertion, intubation, mechanical ventilation, etc.) and hemodynamic support (any blood product transfusion or vasopressor use).

Specific organ injury diagnoses (traumatic brain injury, spinal cord injury, vertebral column injury, thoracic injuries, rib fracture, flail chest, upper extremity injuries, femur fracture, pelvic fracture, lower extremity injuries, and internal organ injuries) and hospital procedure variables were created using the ICD‐10 codes. Preexisting comorbid conditions included cardiac diseases (congestive heart failure, congenital heart disease, and angina pectoris), diabetes, pulmonary diseases (such as chronic obstructive pulmonary disease), coagulopathic disorder (anticoagulant use, bleeding disorder), renal disease, hepatic disease (cirrhosis), and cancer (metastatic cancer, chemotherapy use). Additionally, a binary variable was created to indicate the presence of any of the comorbidities mentioned above.

### Statistical analysis

#### Handling of missing data

To mitigate the risk of bias associated with complete case analysis, we addressed missing vital signs data through multiple imputation. This was achieved by employing a sequential regression method within a multivariate framework, utilizing the fully conditional specification (FCS) option in SAS PROC MI.^23^ Although 20% (*n* = 13,468) of patients had some missing information, the extent of missingness varied across variables, with the highest being BMI (12.4%), which was not retained in the final model. Other variables had considerably lower rates of missingness including GCS (5.7%), RR (3.7%), SBP (2.7%), HR (2.6%), and ISS (0.2%). The sequential regression approach was well suited to this pattern of missing data, as it allowed for variable‐specific imputation models and incorporated all available data to preserve statistical power while minimizing potential biases introduced by excluding cases with missing values. This approach ensured that the imputed values were consistent with the observed data structure, mitigating the impact of missingness on the analysis.

#### Prediction modeling

##### Variable selection

We employed random forest (RF) and classification and regression trees (CART) machine learning methodologies to select and assess potential interaction among predictor variables in developing a modified GTOS. CART is a nonparametric recursive partitioning method that handles data without deleting missing observations. It involves creating a single decision tree through recursive partitioning of the data. In contrast, RF is an ensemble learning method that builds multiple decision trees and combines their outputs to improve predictive performance and robustness against overfitting.^24^ First, using the RF analysis, statistically important covariates were ranked based on variable importance measure (VIM^25^; Figure S1). After selecting variables with relatively high VIM scores from the RF model, we used a single CART‐based decision tree to assess the interaction among the selected predictors. The variables were chosen based on their ranked importance in the RF model, focusing on those with the highest importance scores without applying a strict cutoff.

The final selection of variables was based on statistical importance, measured by the VIM and significant association (*p* < 0.05) with in‐hospital mortality in univariate logistic regression analysis, along with the clinical feasibility of obtaining the information early for risk stratification. For example, highly predictive variables requiring intensive diagnostic workups (e.g., traumatic brain injury) and hospital procedures most likely to be performed after initial risk stratification (orthopedic and nonorthopedic surgical procedures) were intentionally excluded, as this would defeat the purpose of a prognostic model designed to identify patients for early targeted interventions. Multicollinearity between predictor variables was assessed using the variance inflation factor (VIF) and correlation coefficient assessment, ensuring that no prediction variables in the final model were highly correlated.

Subsequently, a predictive model was developed using multivariable logistic regression analysis. The model's discriminative ability between outcome groups was evaluated using the C‐statistic. Calibration of the model was assessed by estimating the misclassification error rate, which quantifies the proportion of incorrect predictions, and the Brier score, which measures the mean squared difference between predicted probabilities and actual outcomes. A Brier score closer to 0 indicates a well‐calibrated model, thereby enhancing the reliability of our prognostic score.^26^

##### Internal validation and risk score generation

The final prediction model underwent internal validation through nonparametric analysis, employing 500 bootstrap samples for robustness. Bootstrapping is widely regarded as a superior approach to validation compared to data‐splitting methods, as it allows the use of the entire data set for model development while providing robust estimates of performance.^27^, ^28^, ^29^ This approach resamples the data set with replacement such that every validation sample has the same number of observations as the full data set, simulating the model's behavior across different samples and accounting for potential overfitting. Unlike data splitting or K‐fold cross‐validation, bootstrapping ensures that validation is performed without reducing the effective sample size. The coefficients of the final model were adjusted for optimism, which means correcting for the tendency of a model to appear more accurate in the development data set than it will be for future patients. This adjustment ensures that the reported performance reflects how the model is likely to perform in new, unseen data.

The modified GTOS score was formulated using the methodology from the Framingham study.^30^ This approach involves selecting a base coefficient from one of the predictors and normalizing the remaining coefficients against this base to assign appropriate points for each patient characteristic.

#### Assessment of clinical utility

**FIGURE 2:**
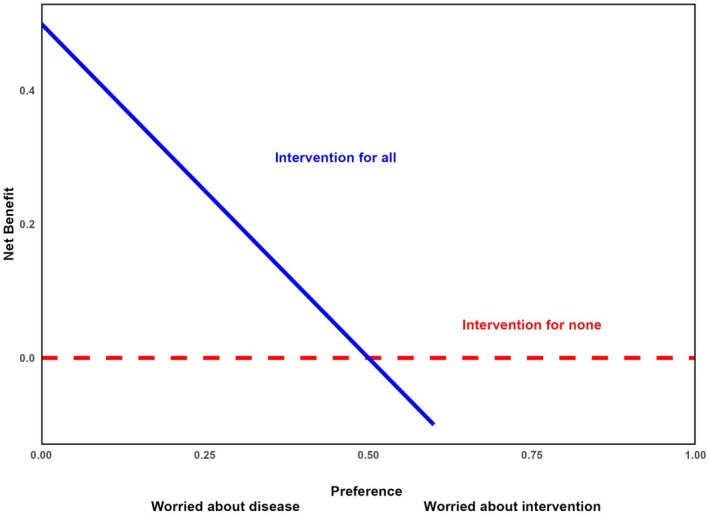
A generic DCA plot. DCA, decision curve analysis.

While AUC analysis can be used to assess model performance, it does not provide information about the clinical utility of a prediction model. We used NB estimation and DCA to evaluate the clinical utility of the prediction models. This method assesses the benefit of using a prediction model in identifying patients at high mortality risk against two default strategies: “Treat all or intervention for all” and “Treat none or intervention for none” (Figure 2).

Figure 2 represents a generic DCA plot. The Y‐axis represents the NB across threshold probabilities for different strategies. The NB cannot exceed the proportion of true outcomes. The X‐axis reflects the treatment threshold probability (Pt), indicating the clinician's tolerance for treating low‐risk patients to capture a high‐risk geriatric trauma patient.

The “treat‐all” curve starts positively but declines, crossing zero when the threshold matches the outcome incidence, as the cost of treating false positives outweighs the benefit of true positives. The “treat‐none” strategy consistently yields zero NB, serving as a baseline.

Central to DCA is the concept of treatment Pt,^31^ representing the point where treatment benefits equal costs. In simpler terms, it is the point at which a clinician is indifferent between treating and not treating a patient based on the predicted risk of an outcome. This threshold reflects the clinician's preference for that type of patient. Clinician preferences for specific patients vary. For instance, setting a threshold at 5% suggests clinicians are willing to treat 19 low‐risk individuals unnecessarily to identify one high‐risk patient. In contrast, a 60% threshold indicates a preference for minimizing false positives, where only patients with a 60% or higher predicted risk are treated, leading to fewer unnecessary interventions.

Risk‐averse clinicians may prefer lower thresholds to take action on minimal mortality risk, while those focused on avoiding unnecessary costs or side effects lean toward higher thresholds. The balance between sensitivity and specificity determines the curve shape, reflecting trade‐offs between false positives and true positives. For example, at higher thresholds, fewer false positives are accepted, but this also means fewer true positives are identified, with significant cost implications.

NB quantitatively evaluates strategies by integrating true positives and false positives with a weighting factor. The NB formula is^18^

$$\mathrm{Net}\;\text{benefit}\left(\mathrm{NB}\right)=\left(\frac{\mathrm{TP}}{N}\right)–\left(\frac{\mathrm{FP}\times \mathrm{Pt}}{N\times \left(1-\mathrm{Pt}\right)}\right),$$

where TP and FP represent true and false positives, Pt is the treatment threshold probability, and *N* is the sample size. NB peaks when no false positives occur or when Pt approaches zero, reflecting maximum benefit. Conversely, when FP costs exceed benefits, NB decreases and can become negative at high thresholds.^31^

We plotted the value of NB yielded by the original GTOS and modified GTOS model against a full range of P_t_ (clinician preferences) to draw decision curves. We then compared the decision curves of GTOS and modified GTOS models against two default decision (treatment) strategies: treat all (considering everyone at high risk of death) and treat none (assuming no one at high risk of death). A model with the highest NB across the range of clinically meaningful threshold probabilities (or at a certain threshold odds under consideration) is deemed the most clinically useful.^17^, ^18^, ^19^

This study received institutional board approval with a waiver of informed consent. The TRIPOD+AI guidelines were followed to ensure proper reporting.^32^ We used SAS 9.4 (SAS Institute Inc.) and Stata 18 (StataCorp LLC) software for analyses. The level of significance was set at *α* = 0.05 (two‐tailed).

## RESULTS

Of the 67,222 geriatric trauma patients admitted to Level I trauma centers, 5.6% died in the hospital. The mean (±SD) age and ISS were 76.7 (±7.3) years and 9.5 (±7.6), respectively. About 3.2% of patients received a pRBC transfusion within 24 h of admission. Table 1 summarizes the demographic and injury characteristics of geriatric trauma patients according to outcome status. Notably, compared to survivors, patients who died in the hospital were older (77.4 years vs. 76.7 years), had higher ISS (22.2 vs. 8.8), utilized more pRBC transfusions (23.1% vs. 2.1%), were predominantly male (62.6% vs. 43.9%), had more penetrating injuries (6.3% vs. 1.6%), and had poorer vital signs, including higher SI (0.7 vs. 0.6) and lower GCS score (9.2 vs. 14.6). Patients who died also underwent more airway interventions performed (61.0% vs. 5.9%).

**TABLE 1:** Patient demographic, etiology, initial ed vital signs, and interventions and procedures by patient outcome.

Characteristics	*N* = 67,222 (%)	In‐hospital mortality		Std. diff.
		Alive (*n* = 63,461)	Died (*n* = 3761)	
Age (years)				
Mean (±SD)	76.7 (±7.3)	76.7 (±7.3)	77.4 (±7.2)	0.15
Median (IQR)	77.0 (70.0–83.0)	77.0 (70.0–83.0)	78.0 (71.0–84.0)	
Age group ≥75	38,874 (57.8)	36,552 (57.6)	2322 (61.7)	−0.13
ISS				
Mean (±SD)	9.5 (±7.6)	8.8 (±6.3)	22.2 (±14.1)	1.27
Median (IQR)	9.0 (4.0–10.0)	9.0 (4.0–10.0)	24.0 (10.0–29.0)	
ISS categories				
<9	28,429 (42.3)	27,998 (44.1)	431 (11.5)	0.79
9–15	28,178 (41.9)	27,354 (43.1)	824 (21.9)	0.46
16–24	6422 (9.6)	5756 (9.1)	666 (17.7)	−0.26
25 or more	4193 (6.2)	2353 (3.7)	1840 (48.9)	−1.2
Any pRBC	2166 (3.2)	1298 (2.1)	868 (23.1)	−0.66
Male	30,189 (44.9)	27,836 (43.9)	2353 (62.6)	−0.37
White race	53,993 (80.3)	51,031 (80.4)	2962 (78.8)	0.02
BMI >35	6316 (9.4)	5990 (9.4)	326 (8.7)	0.02
Penetrating type	1237 (1.8)	1000 (1.6)	237 (6.3)	−0.21
Injury mechanism				
Falls	49,790 (74.1)	47,645 (75.1)	2145 (57.0)	0.29
Gunshot wound/stabbing/assault	2181 (3.2)	1929 (3.0)	252 (6.7)	−0.14
MVC	11,051 (16.4)	9989 (15.7)	1062 (28.2)	−0.24
Other	2705 (4.0)	2500 (3.9)	205 (5.5)	−0.05
Other traffic injuries	1495 (2.2)	1398 (2.2)	97 (2.6)	−0.01
Prehospital EMS transport	55,795 (83)	52,204 (82.3)	3591 (95.5)	−0.42
Initial hospital vital signs				
SBP				
Mean (±SD)	147 (±30.8)	148 (±28.4)	126.2 (±53.8)	−0.28
Median (IQR)	147 (128.0–165.0)	147 (130.0–165.0)	133 (101.0160.0)	
SBP ≤110 or ≥ 200	6280 (9.3)	5128 (8.1)	1152 (30.6)	−0.44
HR				
Mean (±SD)	82.0 (±18.3)	81.9 (±16.8)	82 (±34.2)	0.34
Median (IQR)	80.0 (70.0–92.0)	80.0 (70.0–91.0)	84.0 (68.0–101.0)	
HR ≤60 or ≥110	9896 (14.7)	8610 (13.6)	1286 (34.2)	−0.36
SI				
Mean (±SD)	0.6 (±0.4)	0.6 (±0.2)	0.7 (±1.6)	0.14
Median (IQR)	0.6 (0.5–0.7)	0.5 (0.5–0.7)	0.6 (0.5–0.8)	
SI >0.9	3616 (5.4)	2940 (4.6)	676 (18.0)	−0.47
RR				
Mean (±SD)	18.2 (4.0)	18.2 (3.7)	17.7 (7.3)	−0.23
Median (IQR)	18.0 (16.0–20.0)	18.0 (16.0–20.0)	18.0 (16.0–20.0)	
RR ≤10 or ≥24	5060 (7.5)	4122 (6.5)	938 (24.9)	−0.44
Total GCS				
Mean (±SD)	14.3 (±2.2)	14.6 (±1.4)	9.2 (±5.2)	−1.31
Median (IQR)	15.0 (14.0–15.0)	15.0 (14.0–15.0)	10.0 (3.0–15.0)	
GCS ≤13	5392 (8.0)	3105 (4.9)	2287 (60.8)	−1.37
Procedures and interventions				
Airway intervention	6027 (9.0)	3734 (5.9)	2293 (61.0)	−1.51
Hemodynamic support	7894 (11.7)	6631 (10. 5)	1263 (33.6)	−0.61
Initial ED supplemental oxygen	9689 (14.4)	7739 (11.5)	1950 (51.9)	−0.91

*Note*: Data are reported as *n* (%) unless otherwise specified. Airway intervention includes invasive and noninvasive airway support, including airway insertion, intubation, and mechanical ventilation.

Abbreviations: BMI, body mass index; HR, heart rate; GCS, Glasgow Coma Scale; ISS, Injury Severity Score; MVC, motor vehicle collision; RR, respiratory rate; SBP, systolic blood pressure; SI, Shock Index; Std. diff., standardized difference (calculated: died–alive).

Table 2 summarizes the patients' injury characteristics and preexisting comorbidities. Patients who died had more head/face (75.2% vs. 44.5%), chest (41.5% vs. 21.6%), and abdominal injuries (19.8% vs. 6.3%) but fewer extremity (63.2% vs. 74.0%) injuries than patients who survived. Expectedly, among patients who died, there was a higher incidence of multiple injuries (55.8% vs. 35.2%) and torso injuries (54.2% vs. 34.1%). Additionally, there was a higher prevalence of cardiac diseases (11.9% vs. 8.5%), coagulopathic disorder (24.5% vs. 20.9%), renal diseases (5.7% vs. 3.4%), hepatic diseases (2.7% vs. 1.1%), and metastatic cancer (3.2% vs. 2.0%) among patients who died in hospital than patients who survived. The proportion of patients with any of the cardiac, hepatic, or coagulopathic comorbidities was higher among those who died in the hospital (32.1% vs. 26.4%) compared to those who survived.

**TABLE 2:** Injury characteristics and comorbidity by patient outcomes.

Characteristics	*N* = 67,222 (%)	In‐hospital mortality		Std. diff.
		Alive (*n* = 63,461)	Died (*n* = 3761)	
Injured body region				
Head/face	31,091 (46.3)	28,264 (44.5)	2827 (75.2)	−0.66
Chest	15,294 (22.8)	13,735 (21.6)	1559 (41. 5)	−0.38
Abdomen	4722 (7)	3976 (6.3)	746 (19.8)	−0.37
Extremities	49,358 (73.4)	46,983 (74.0)	2375 (63.2)	0.24
Multiple injuries	24,404 (36.3)	22,306 (35.2)	2098 (55.8)	−0.39
Injury to torso	23,661 (35.2)	21,621 (34.1)	2040 (54.2)	−0.38
Specific injuries (ICD‐10)				
Traumatic brain injury	7650 (11.4)	6474 (10.2)	1176 (31.3)	−0.54
Spinal cord injury	987 (1.5)	804 (1.3)	183 (4.9)	−0.2
Vertebral column injury	12,012 (17.9)	10,938 (17.2)	1074 (28.6)	−0.28
Thoracic	3233 (4.8)	2398 (3.8)	835 (22.2)	−0.54
Rib fractures	12,387 (18.4)	11,178 (17.6)	1209 (32.2)	−0.31
Flail chest	551 (0.8)	409 (0.6)	142 (3.8)	−0.2
Upper extremity injury	20,004 (29.8)	18,752 (29.6)	1252 (33.3)	−0.07
Femur fracture	15,007 (22.3)	14,488 (22.8)	519 (13.8)	0.23
Pelvic fracture	5437 (8.1)	4909 (7.7)	528 (14.0)	−0.19
Other lower extremity injuries	16,568 (24.6)	15,596 (24.6)	972 (25.9)	0.01
Internal organ injuries	6155 (9.2)	5119 (8.1)	1036 (27.6)	−0.48
Preexisting comorbidities				
Any comorbidities	26,828 (39.9)	25,024 (39.4)	1804 (48.0)	0.19
Cardiac disease	5843 (8.7)	5398 (8.5)	445 (11.9)	−0.14
Diabetes	17,105 (25.4)	16,213 (25.6)	892 (23.7)	0
Pulmonary	7531 (11.2)	7083 (11.2)	448 (11.9)	−0.05
Coagulopathic disorder	14,185 (21.1)	13,265 (20.9)	920 (24.5)	−0.13
Renal disease	2357 (3.5)	2142 (3.4)	215 (5.7)	−0.13
Hepatic disease	824 (1.2)	724 (1.1)	100 (2.7)	−0.12
Cancer	1413 (2.1)	1291 (2.0)	122 (3.2)	−0.09
Cardiac/hepatic/coagulopathic	17,951 (26.7)	16,744 (26.4)	1207 (32.1)	−0.17

*Note*: Data are reported as *n* (%). Injury to the torso region includes any injury to the chest or abdomen. Std. diff. greater than ±0.1 was considered clinically meaningful.

Abbreviations: Std. diff., standardized difference (calculated: died–alive).

### Multivariable analysis and modified GTOS

The RF method ranked the predictor variables based on their relative importance using the VIM. Figure S1 presents the variable ranking according to the VIM. The final selection of variables was determined by their statistical significance and the clinical feasibility of obtaining the information early for risk stratification. Statistical significance was assessed using the *p*‐values from the logistic regression analysis performed after the RF classification to confirm the importance of the variables identified by VIM. A single tree‐based CART method identified no interaction among the final predictor variables selected for prognostic risk scoring. The seven variables incorporated in the final multivariable logistic regression model in descending order of prognostic importance were: initial ED GCS score of ≤13, any airway support, pRBC transfusion within 24 h, penetrating injury, age ≥75 years, preexisting comorbidities, and injury to the torso region. The most frequent airway procedure was insertion of endotracheal tube (endoscopic) followed by endoscopic draining of pleural cavity with drainage device (Table S1). Preexisting comorbidity was measured as the presence of any of the following comorbid conditions: cardiac diseases, coagulopathy disorder, or hepatic diseases. These particular comorbidities were identified as important predictors by the RF analysis. Patients who died with torso injuries (2024 of 23,661) predominantly had chest injuries (76.4%), including 59.2% with rib fractures (Table S2).

A risk score was created by assigning weights based on the coefficients of these seven binary variables, and its performance was assessed using univariate logistic regression analysis with the risk score as the sole predictor of in‐hospital mortality. Table 3 details the variables and their contribution to the risk score. The modified GTOS ranges from 0 to 19, with GCS ≤13 and any airway intervention being the strongest predictors. Notably, 17.2% of patients had a risk score of zero. A risk score of 5 maximized sensitivity and specificity, achieving values of 0.84 and 0.90, respectively, with a predicted probability of 0.058.

**TABLE 3:** The modified GTOS: Predictors of in‐hospital mortality among injured older adults.

Predictor variables	Adjusted OR (95% CI)	Coefficient	Score contribution
GCS≤13	12.2 (11.1–13.3)	2.498	5
Airway intervention	8.9 (8.1–9.8)	2.19	5
RBC transfusion within 24‐h	3.3 (2.9–3.8)	1.187	3
Penetrating injury	2.5 (2–3.1)	0.9	2
Age ≥75	2 (1.9–2.2)	0.716	2
Preexisting comorbidities	1.8 (1.6–2)	0.584	1
Injury to the torso region	1.6 (1.5–1.8)	0.481	1
Intercept		−5.03	
Maximum possible score			19

*Note*: Selection informed by random forest and classification and regression tree methods; age, pRBC: original GTOS model. Airway intervention includes invasive and non‐invasive airway support, including airway insertion, intubation, and mechanical ventilation. Preexisting comorbidity represents the presence of any cardiac diseases, hepatic diseases, and coagulopathic disorders (including anticoagulant use). Injury to the torso region includes any injury to the chest or abdomen.

Abbreviation: GTOS, Geriatric Trauma Outcome Score.

**FIGURE 3:**
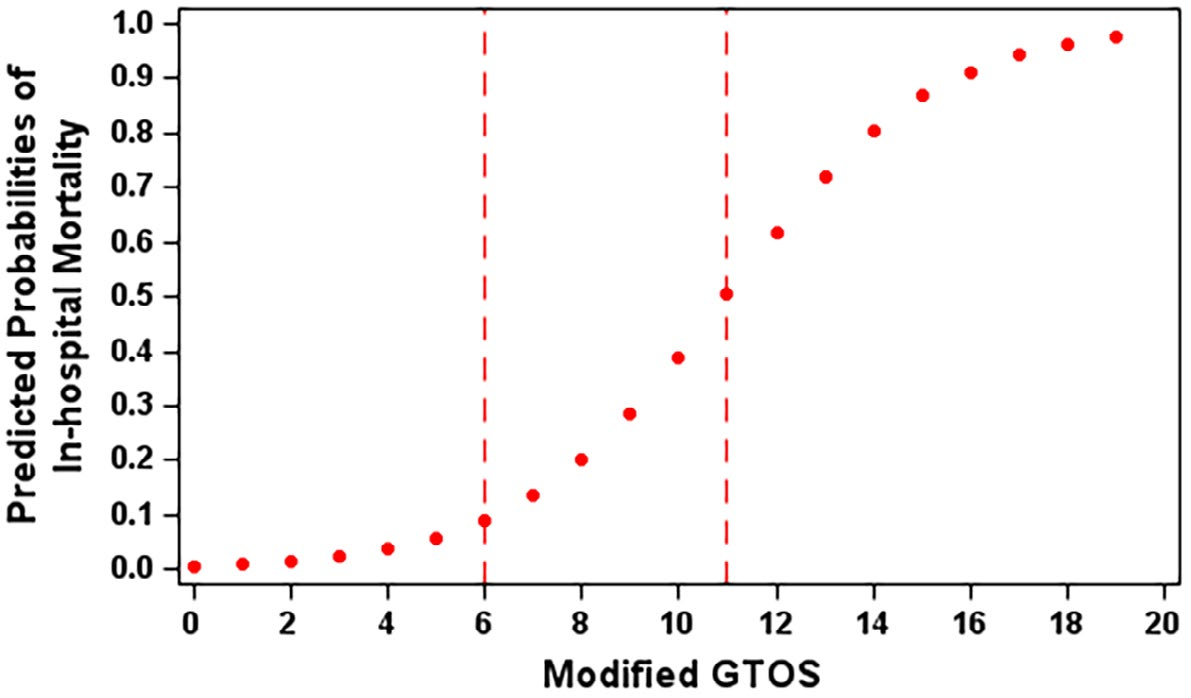
Predicted probability of in‐hospital mortality, the modified GTOS model. GTOS, Geriatric Trauma Outcome Score.

Using the modified GTOS, clinicians can calculate an absolute probability of in‐hospital mortality. For example, a patient aged ≥75 years, with a GCS ≤13, with a penetrating injury, and who also received pRBC transfusion, would have a risk score of 12, corresponding to a predicted probability of in‐hospital mortality of 62% as shown in Figure 3.

The recursive partition analysis revealed that risk scores of 6 and 11 define the medium‐ and high‐risk groups, respectively, with predicted outcome probabilities of 0.27 and 0.59 at these thresholds. The observed incidence of mortality for the low‐, medium‐, and high‐risk categories was 1.25%, 26.64%, and 58.55%, respectively.

In a univariate logistic regression model where the modified GTOS was the sole predictor of in‐hospital mortality, it demonstrated a significantly higher AUC (AUC 0.92, 95% CI 0.91–0.92) compared to the GTOS model (AUC 0.84, 95% CI 0.83–0.85, *p* < 0.0001). Additionally, the modified GTOS had a lower misclassification error rate (4.9% vs. 5.2%) and lower Brier score (0.036 vs. 0.042) than the GTOS model as shown in Table 4.

**TABLE 4:** Comparison of calibration and discrimination indicators for GTOS and modified GTOS model.

Parameter	GTOS model	Modified GTOS model
Calibration		
Misclassification error	5.2%	4.9%
Brier score	0.042	0.036
Discrimination		
AUC	0.84 (95% CI 0.83–0.85)	0.92 (95% CI 0.91–0.92)

*Note*: Misclassification error rate quantifies the proportion of incorrect predictions. Brier score measures the mean squared difference between predicted probabilities and actual outcomes. A Brier score closer to 0 indicates a well‐calibrated model.

Abbreviations: AUC, area under the receiver operating characteristic score; GTOS, Geriatric Trauma Outcome Score model.

### Assessment of clinical utility

**FIGURE 4:**
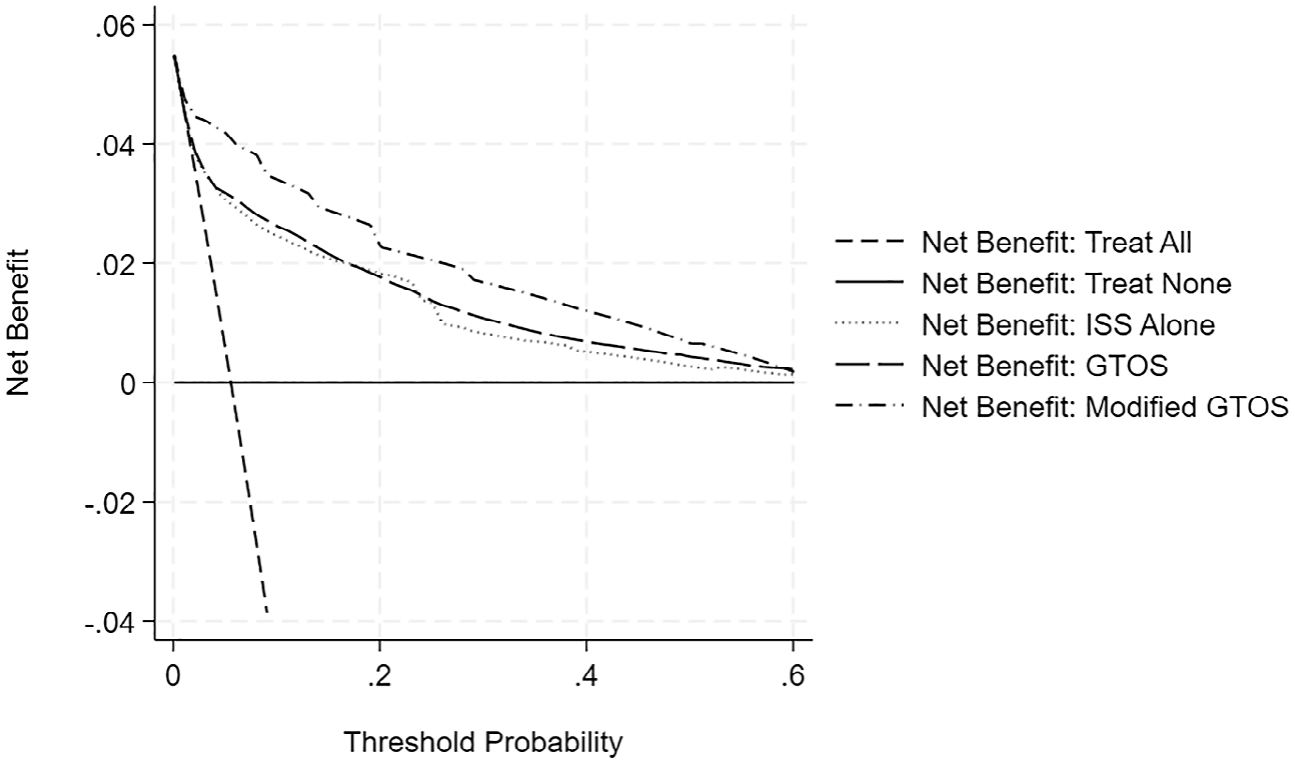
DCA comparing prediction of in‐hospital mortality Based on GTOS and modified GTOS models. DCA, decision curve analysis; GTOS, Geriatric Trauma Outcome Score.

The DCA revealed that the modified GTOS provides a higher NB than a decision based on strategies: treat all, treat none, and treat those who are predicted at high mortality risk by the original GTOS model across a wide range of treatment threshold probabilities as it lies above all other curves, as shown in Figure 4. The modified GTOS model and the GTOS model showed no benefit when used beyond a 60% risk threshold, which is more than 10 times the average predicted risk of 5.6%. It is important to note that the purpose of DCA is to aid clinicians in making decisions in situations where they are undecided between treating all patients or treating none.

## DISCUSSION

The modified GTOS model had a higher AUC (0.92 vs. 0.83), lower misclassification error rate (4.9% vs. 5.2%), and Brier score (0.036 vs. 0.042) than the GTOS model. These differences were statistically significant (*p* < 0.05) and clinically meaningful. The incidence of in‐hospital mortality among geriatric trauma patients treated at Level I trauma centers was 5.6%. NB estimation and DCA found that, except at very high‐risk thresholds (>10 times the average risk of 5.6%), intervening on patients based on the modified GTOS model led to improved clinical outcomes compared to intervening in all patients, intervening in no patients, or intervening on those who are predicted with high mortality risk by the GTOS model.

The GTOS consists of age, ISS, and transfusion of any pRBC within 24 h of injury. It is widely used in the prediction of in‐hospital mortality among geriatric trauma patients.^33^, ^34^, ^35^, ^36^ Since this study aimed to modify the GTOS to improve clinical usefulness, we excluded ISS from the model because it often requires time‐consuming diagnostic radiological interventions, making it unsuitable for early risk stratification.^37^, ^38^ The GTOS model used age as a continuous predictor, but the modified model categorized age using a cutoff of 75 years. A systematic review reported that trauma‐related mortality rate stabilizes after the age of 74 years, without any further increase.^39^ Using a binary variable simplifies estimating and interpreting the risk score. The pRBC transfusion within 24 h of admission was considered a priori selection for the modified model.

Modification of the original GTOS model included the addition of the following clinical characteristics: low initial ED GCS (GCS ≤13), early airway interventions, injury type, existing comorbidities, and torso injury. Low GCS (GCS ≤13) is an established predictor of trauma mortality.^22^, ^40^, ^41^, ^42^ The GCS estimation is relatively straightforward and can be assessed quickly. It evaluates three components: eye opening, verbal response, and motor response. Additionally, it can be performed at the bedside without additional information or imaging. The authors who validated the GTOS model acknowledged that the GCS could be associated with mortality and considered its exclusion a limitation of their study.^10^

Airway intervention is an independent predictor of mortality or severe trauma among geriatric trauma,^22^, ^43^ and this was identified as an important predictor in our modified GTOS. Likewise, although most geriatric trauma patients present with blunt injuries, the risk of mortality is significantly higher among those with penetrating.^44^

While the original GTOS model did not consider existing comorbidities, our modified GTOS model identified them as a strong predictor of in‐hospital mortality. Previous studies highlighted the significance of comorbidities in trauma outcomes and recommended that prediction models should incorporate patients’ chronic comorbidities.^45^, ^46^ Important preexisting comorbid conditions, identified through the RF and classification and regression tree analysis, included cardiac disease, hepatic disorders, and coagulopathic disorders (including anticoagulant use), which were incorporated into the modified GTOS model.

Our modified GTOS model includes torso injuries as a predictor of in‐hospital mortality. The torso, containing vital organs, can present life‐threatening complications if injured. By accounting for chest injuries, which represent 10% of trauma admissions and about a quarter of trauma‐related fatalities,^47^ and abdominal injuries, which are the third most common injury requiring major surgical intervention in 25% of cases,^48^ the model enhances its predictive capacity. Torso injuries can be rapidly assessed with minimal diagnostic intervention, making them a valuable predictor for early risk stratification in injured geriatric patients.

The variables included in the modified model were selected using machine learning methods. One could argue that the modified GTOS model showed higher discrimination (i.e., AUROC) than the GTOS model because it incorporates more variables. While this is partially true, the variables included in the modified GTOS model can be measured or identified earlier in the hospital course of a patient, unlike the ISS, which generally requires time‐consuming diagnostic imaging tests to establish. This approach aligns with the understanding that early and accurate prediction of mortality risk in geriatric trauma patients can significantly impact clinical outcomes through early recognition of patients at high risk for adverse outcomes. By focusing on variables that can be assessed promptly upon admission, the modified GTOS model facilitates quicker decision making and timely interventions, potentially improving survival rates.

Prior studies have mainly used the AUC to measure and compare the accuracy of prediction models. One limitation of AUC is that it fails to incorporate the cost of misclassification error. For example, two models—one with high specificity and lower sensitivity and another with higher sensitivity than specificity—could show similar or exact AUC, but their clinical usefulness may differ. In trauma care, the cost of a false negative—failing to identify a high‐risk patient—can be catastrophic, leading to missed opportunities for lifesaving interventions. Therefore, the sensitivity–specificity trade‐off is uneven, and a model with high sensitivity is preferred over a model with high specificity but low sensitivity.

This study is the first to evaluate a trauma outcome prediction model employing DCA and NB assessment. NB directly reflects the clinical utility of a model by quantifying the balance between beneficial and harmful decisions, thus offering a more practical perspective.^17^, ^18^, ^19^, ^20^ The NB is particularly advantageous in trauma settings where misclassification costs can be significant, guiding clinical decision making more effectively than AUC alone. Additionally, the DCA helps incorporate the misclassification cost at different risk thresholds for patients, allowing for a more nuanced evaluation, considering varying risk tolerances or preferences and the potential impact on patient outcomes. It further allows us to compare the clinical utility of different decision strategies for a patient at various risk thresholds and helps identify the best intervention strategy at varying risk thresholds. The original GTOS and our modified GTOS models showed no benefit when used beyond a 60% risk threshold, which is more than 10 times the average predicted risk (5.6%). It is essential to consider that the purpose of the DCA is to help clinicians make decisions where they are indifferent in deciding to treat all or none. While we do not have any empirical data to justify the threshold beyond which clinicians do not require additional tools to make decisions regarding interventions for trauma patients, a risk threshold more than 10 times the average probability seems reasonable. For example, the preference level or risk threshold for using a CT in severely injured patients was considered 17:1, meaning that clinicians prefer to perform 17 CT scans to save one life.^49^ In this scenario, the risk threshold for decision making is only 5.6%.

The increasing incidence of geriatric trauma, coupled with the complexity of care required for this vulnerable population, underscores the necessity for precise and timely risk stratification. The GTOS model has been a valuable tool, originally designed to predict in‐hospital mortality using age, ISS, and blood transfusion within 24 h of admission.^10^, ^50^ The GTOS model is already externally validated in a multicenter study^10^ and studied in diverse contexts.^33^, ^34^, ^35^, ^36^ However, the GTOS model's reliance on ISS, which often necessitates advanced imaging and time‐consuming procedures, limits its utility for early predictions. The modified GTOS model primarily focuses on predictors available within the first 24 h to stratify the risk of in‐hospital mortality. The interventions that are typically considered in trauma settings, such as airway management, blood transfusions, and surgeries, are often standardized based on clinical protocols and the patient's initial presentation. The risk threshold discussed in the DCA guides whether to intervene based on the predicted risk. The assumption is that higher risk patients identified by the model would receive more intensive monitoring and possibly earlier interventions. However, it is essential to recognize that the DCA does not dictate specific interventions but helps decide whether the predicted risk justifies intervention. In practice, interventions’ exact nature and extent would depend on clinical judgment and available resources. Future studies should aim to detail specific interventions associated with different risk scores to clarify this aspect further.

## LIMITATIONS

This study has some limitations that are worth further explanation. It is based on retrospective data from institutions participating in the TQIP, which may not represent all trauma care facilities. Centers more actively engaged in quality improvement initiatives might report better outcomes, potentially skewing the results. Limiting the study population to geriatric trauma patients directly admitted to Level I trauma centers was a deliberate selection criterion, ensuring that the modified GTOS model is used in an environment where high‐standard, timely interventions are possible. This setting provides an optimal scenario for assessing the model's potential predictive accuracy and clinical utility. However, it is important to note that this focus may limit the generalizability of the results to other settings with fewer resources. Patients in Level I centers often receive comprehensive care that may not be available in lower level trauma centers, potentially affecting the model's performance in those environments. Since this is an initial study, future research should aim to validate the model in diverse health care settings to ensure its broader applicability and effectiveness in guiding clinical decision making for geriatric trauma patients across different trauma care contexts.

## CONCLUSIONS

This study demonstrates that the predictive accuracy of the Geriatric Trauma Outcome Score model can be significantly improved by replacing the Injury Severity Score with clinical characteristics that are available for early risk stratification, such as initial hospital airway interventions, ED Glasgow Coma Scale score, preexisting comorbidities, injury type, and the presence of torso injuries. By leveraging decision curve analysis and net clinical benefit analysis, the study highlights the enhanced clinical utility of the modified Geriatric Trauma Outcome Score model, enabling more informed and timely clinical decisions. Future research should focus on further validation and practical implementation to fully harness the benefits of this improved predictive model. Aligning predictive efforts with the realities of clinical decision making has the potential to provide timely care and improve patient outcomes.

## AUTHOR CONTRIBUTIONS

Conception and study design: Pawan Acharya, Tabitha Garwe. Literature review: Pawan Acharya. Data acquisition: Pawan Acharya, Tabitha Garwe. Data analysis and interpretation: Pawan Acharya, Tabitha Garwe, Sara K. Vesely. Drafting of the manuscript: Pawan Acharya, Tabitha Garwe. Critical revision: Pawan Acharya, Tabitha Garwe, Sara K. Vesely, Amanda Janitz, Jennifer D. Peck, Alisa M. Cross.

## FUNDING INFORMATION

Partial funding provided by the National Institutes of Health, National Institute of General Medical Sciences (Grant 1 U54GM104938), an IDeA‐CTR to the University of Oklahoma Health Sciences Center.

## CONFLICT OF INTEREST STATEMENT

The authors declare no conflict of interest. I, Pawan Acharya, attest on behalf of all authors, that we had full access to the data of the study, conducted all data analyses independently from the funding entity, and take complete responsibility for the integrity and accuracy of the data reported in the manuscript.

## Supporting information


Figure S1.
Table S1.
Table S2.
Text S1.

## Data Availability

The data used in this study were obtained from the American College of Surgeons (ACS) Trauma Quality Improvement Program (TQIP) and are not publicly available. Access to TQIP data requires formal application and approval by the ACS. Interested researchers can find more information about the application process and data use policies at: https://www.facs.org/quality‐programs/trauma/quality/national‐trauma‐data‐bank/datasets/.
