# Supplementary material for: Enhancing geriatric trauma mortality prediction: Modifying and assessing the Geriatric Trauma Outcome Score with net benefit and decision curve analysis
Source: Acad Emerg Med. 2025 Feb 6;32(6):668–80. doi: 10.1111/acem.15103 (PMC12171684; doi:10.1111/acem.15103)
Supplement: Supplementary file 1 — Figure S1. Table S1. Table S2. Text S1. [file ACEM-32-668-s001.zip › Supplemental_Data.docx]

**Supplemental Data**

**Figure S1. Variable Importance Measure Plot for In-hospital Mortality Predictors**


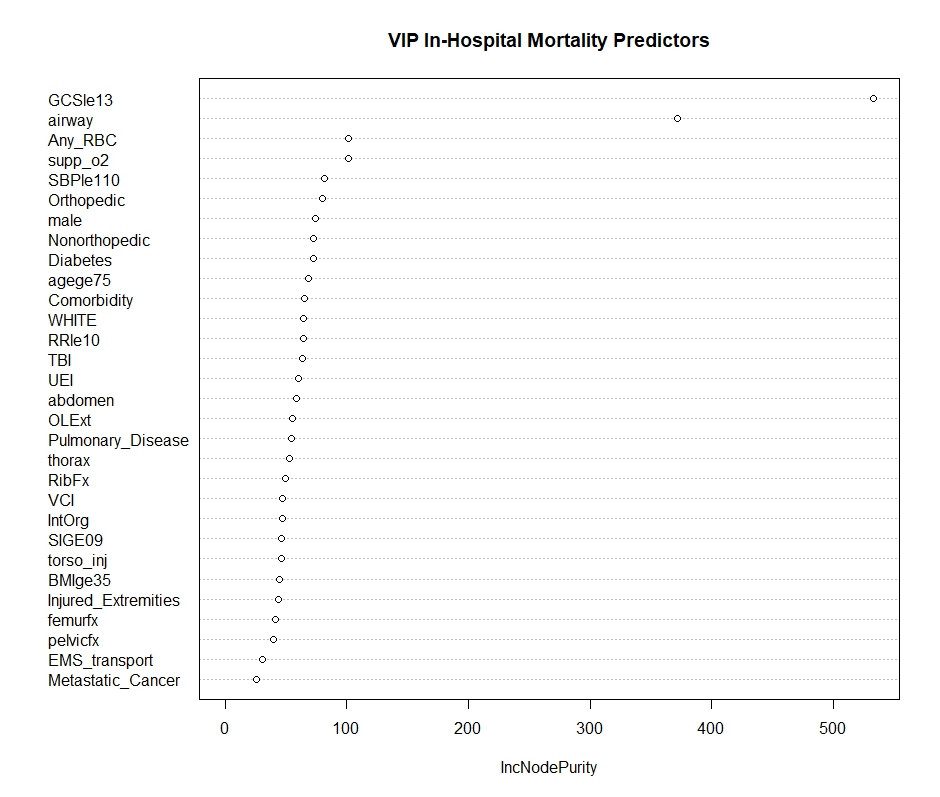


**Table S1. Description of Top Ten Airway Procedures (n=5921)**

| **ICD Procedure Code** | **Procedure Name** | **Percent (%)** |
| --- | --- | --- |
| 0BH17EZ | Insertion of Endotracheal Airway, Endoscopic | 5.4 |
| 0T9B70Z | Drainage of Pleural Cavity with Drainage Device, Endoscopic | 1.44 |
| 0BJ08ZZ | Inspection of Trachea, Via Natural or Artificial Opening, Endoscopic | 0.68 |
| 0BH18EZ | Insertion of Endotracheal Airway, Endoscopic | 0.64 |
| 0DH63UZ | Dilation of Trachea, Endoscopic | 0.48 |
| 0B110F4 | Insertion of Nasopharyngeal Airway, Endoscopic | 0.48 |
| 0DJ08ZZ | Inspection of Trachea, Via Natural or Artificial Opening, Endoscopic | 0.21 |
| 0BJ88ZZ | Inspection of Bronchus, Endoscopic | 0.1 |
| 0B9F8ZX | Drainage of Trachea, Endoscopic | 0.08 |
| 0BJH8ZZ | Inspection of Trachea, Percutaneous Endoscopic | 0.07 |

% Represent the share of the listed procedure among total airway procedure.

**Table S2. Description of Patients with Torso Injuries (n= 23661)**

| **Injury Description** | **In-hospital Mortality** | | |
| --- | --- | --- | --- |
|  | **Alive (n=21621)** | **Died (n=2040)** | P-value |
| **Chest torso injury** |  |  |  |
| Any Chest Injury | 13735 (63.53) | 1559 (76.42) | p<0.001 |
| Chest AIS>=3 | 7510 (34.73) | 1120 (54.9) | p<0.001 |
| Rib fracture | 11176 (51.69) | 1208 (59.22) | p<0.001 |
| Flail Chest | 409 (1.89) | 142 (6.96) | p<0.001 |
| **Abdominal injury** |  |  |  |
| Any Abdominal Injury | 3976 (18.39) | 746 (36.57) | p<0.001 |
| Abdomen AIS>=3 | 678 (3.14) | 273 (13.38) | p<0.001 |
| **Internal Organ Injury** | 5118 (23.67) | 1036 (50.78) | p<0.001 |
| Liver | 439 (2.03) | 215 (10.54) | p<0.001 |
| Heart/lung injury | 1828 (8.45) | 530 (25.98) | p<0.001 |
| Spleen Injury | 647 (2.99) | 187 (9.17) | p<0.001 |

AIS: Abbreviated Injury Scale

P-values were calculated using the Chi-squared test.
